# Normal Levels of Urinary CC16 Protein. Comments on Beamer et al. Association of Children’s Urinary CC16 Levels with Arsenic Concentrations in Multiple Environmental Media. *Int. J. Environ. Res. Public Health* 2016, *13*, 521.

**DOI:** 10.3390/ijerph13100977

**Published:** 2016-10-04

**Authors:** Víctor García-Nieto, Domenico Mancini, Eva Rodríguez-Carrasco

**Affiliations:** Hospital Nuestra Señora de Candelaria, Santa Cruz de Tenerife 38010, Spain; domenico_mancini@alice.it (D.M.); evita1809@hotmail.com (E.R.-C.)

In 1937, Max Clara described a new type of cell in the human lung, which was later determined to be an exocrine secretory cell type containing granules composed of proteins [1]. In 1988, in an exploratory study on proteins obtained from lung washes, Singh et al. identified a protein that is expressed in both rodents and humans, which was subsequently named Clara cell 16-kDa protein (CC16) [2]. It constitutes a major protein secreted by Clara cells in the respiratory tract and is secreted to the epithelial lining fluid of the lung and transported up along the bronchiolar tree [3].

Given its small size, with a molecular weight of 15.8 kDa, CC16 is rapidly filtered by the glomerulus, reabsorbed almost entirely, and catabolized in the renal proximal tubule cells. This mechanism is similar to that of β2-microglobulin, cystatin C, and other low-molecular-weight proteins.

A proximal tubular dysfunction results in the diminished resorption of CC16 and increased levels in the urine. Since the early 1990s, the determination of urinary CC16 has sporadically been used as a marker of tubular renal proximal dysfunction [4].

Recently, Beamer et al. have published in this journal an interesting work whose objective was to determine if urinary CC16 levels in children are associated with arsenic concentrations in environmental media collected from their homes [5]. Their hypothesis was based on the fact that arsenic exposure has been associated with decreased CC16 levels in adults. The authors concluded that lower levels of CC16 in children’s urine may be associated with exposure to arsenic via multiple routes.

These results have two controversial aspects. The first one is that the kidney is a known target organ of arsenic toxicity and is critical for both in vivo arsenic biotransformation and elimination. For more than a century, it has been well known that intoxication by arsenic produces vascular or tubular histological lesions for which the term “the arsenic kidney” was created [6]. Later, it was confirmed that chronic arsenic administration in experimental animals originates ultrastructural alterations that consist in swollen mitochondria and increased numbers of dense autophagic lysosome-like bodies in the proximal tubule cells [7]. Moreover, a subcytotoxic dose of arsenite induces necrotic changes in the cytoplasmic structure and mitochondrial morphology in the human proximal tubular cell line [8]. Recently, Tokumoto et al. described that inorganic arsenic may induce p53-dependent apoptotic pathways through the downregulation of gene expression of the *Ube2d* family in proximal tubular cells [9]. With the intention of examining the association between arsenic exposure and renal dysfunction, Chen et al. studied 1043 subjects from a population in Taiwan. The authors observed an abnormal urinary elimination of β2-microglobulin when urinary levels with an arsenic-to-creatinine ratio were above 35 μg/g [10]. In a Mexican study, an association between urinary arsenic levels and α1-microglobulin urinary excretion, another low-molecular-weight protein that is a marker of early tubular proximal renal injury, was observed [11]. Consequently, the patients studied by Beamer et al. [5] should have elevated or at least normal CC16 levels, making difficult to accept that they had reduced levels upon exposure to arsenic.

The second controversial point refers precisely to the reduced urinary levels to which the paper of Beamer et al. refers [5]. Under physiological conditions, the quasi-totality of this protein that is filtered is reabsorbed in the proximal tubule in such a way that the detectable levels in urine are very low.

Therefore, in our first paper on the subject, the value of the urinary CC16-to-creatinine ratio was zero in 16 of the 31 control children (51.6%) [12]. In another recent study by our group [13], the average urinary CC16-to-creatinine ratio in control children (1.12 ± 0.59 μg/g) (*n* = 28) was even lower than that in the first study (1.22 ± 1.52 μg/g).

In summary, we believe that Beamer et al. [5] should reconsider the discussion of the results to which your paper refers given that having low urinary CC16 levels is normal.
